# Less-Lethal Weapons and Civilian Injury in Police Use of Force Encounters: A Multi-agency Analysis

**DOI:** 10.1007/s11524-024-00940-1

**Published:** 2024-11-11

**Authors:** Kevin Petersen, Christopher S. Koper, Bruce G. Taylor, Weiwei Liu, Jackie Sheridan-Johnson

**Affiliations:** 1 Department of Criminal Justice and Criminology, University of North Carolina at Charlotte, Charlotte, NC USA; 2 Department of Criminology, Law and Society, George Mason University, Fairfax, VA USA; 3 Public Health Department, NORC at the University of Chicago, Bethesda, MD USA

**Keywords:** Police, Less-lethal weapons, Use-of-force, Injury

## Abstract

Police use-of-force is a growing public health concern, with recent estimates suggesting that over 70,000 people are injured by police each year. To reduce the risk of injury to civilians, most police agencies authorize the use of various less-lethal weapons. However, to date, there is little consensus as to which types of less-lethal weapons are most effective at reducing injury risk. In this study, we test the differential effects of less-lethal weapons on civilian injury and injury severity using data on 2348 use-of-force incidents originating from 17 large urban and metropolitan law enforcement agencies from 2015 to 2019. Specifically, we assess the injury risks associated with conducted energy devices, chemical agents, impact weapons, and police canines, while controlling for a robust set of officer, civilian, and situational characteristics. Our results indicate that chemical agents reduce the risk of hospitalization or death significantly more than other weapon types, while police canines increase the risk of all injury outcomes significantly more than other weapon types. Adjusting for incident characteristics, chemical agents are predicted to cause hospitalization or death in 4% of cases, compared to 13% for conducted energy devices, 16% for impact weapons, and 37% for police canines. These findings suggest that civilian injury may be reduced through use-of-force policies that prioritize less severe modalities of force, though more research is needed on the contextual and long-term effects of these weapons.

**Supplementary Information:**

The online version contains supplementary material available at 10.1007/s11524-024-00940-1.

## Introduction

Over the past decade, high-profile deaths of civilians in police custody and the use of law enforcement to quell social and political protests have solidified police use-of-force (UOF) as a significant public health issue [1–4]. Though only a small percentage of police-civilian encounters result in the use of force [5, 6], civilian injury in these encounters is relatively common (e.g., 40–50% of incidents) [7, 8]. Recent estimates suggest that over 70,000 people are injured during police encounters annually [9], and lifetime medical expenses from these injuries may surpass $200 million in a single year [10]. To combat this, many law enforcement agencies authorize the use of less-lethal weapons, commonly including chemical agents (e.g., oleoresin capsicum spray, also known as pepper spray), electronic control weapons/conducted energy devices (ECW/CED, e.g., TASERs®), impact weapons (e.g., collapsible batons), and/or police canines [11–13]. Nationally, over 90% of law enforcement agencies employ less-lethal weapons in some capacity [14], and estimates suggest that less-lethal weapons are used in upwards of 20–30% of UOF encounters [6, 11, 15–17].

Though their mechanisms of effect differ, less-lethal weapons are intended to provide police officers with the means to apprehend resistant suspects without the need for hands-on tactics or firearms, thus (in theory) reducing the risk of civilian injury and death [12, 13, 17]. Existing research generally supports this claim, suggesting that less-lethal weapons such as ECW/CEDs and chemical agents reduce civilian injury and death, relative to traditional forms of force [6, 11, 15–20]. However, the rate at which various less-lethal weapons result in civilian injury—along with the severity of these injuries—has not been firmly established. Recent studies indicate that less-lethal weapons such as chemical agents and kinetic impact projectiles can result in serious injury or death, but heterogeneity in sample selection and study design often preclude estimation of injury risk for the larger population of individuals exposed to these weapons [4, 21–23].

In addition, few studies to date have directly compared the injury risks associated with specific forms of less-lethal weapons, and among those that have, findings have been inconsistent [6, 15, 16, 24]. This is a salient issue because most police agencies (e.g., 80% or more) train officers to use force according to an administratively determined continuum that authorizes increasingly severe forms of force as necessary, given the characteristics of an encounter [25]. These UOF continuum or “force option” models designate the type (or types) of force that are justifiable based on the level of suspect resistance and/or threat [24–26]. In practice, however, there is considerable variation in the structure of these models and the order by which agencies rank the severity of less-lethal weapons [14, 24, 25]. Often, for example, agencies rank multiple less-lethal weapons (e.g., chemical agents, ECW/CEDs) within the same level of force [25], providing officers discretion over the choice of weapon used in an encounter. If certain less-lethal weapons pose a greater risk to civilian safety than others, however, this discretion could lead to the disparate application of force and risk of injury across otherwise comparable encounters.

Several studies suggest that, in general, ECW/CEDs and chemical agents are less likely to cause injury than police canines and impact weapons [6, 11, 15–17]. However, the relationship between ECW/CEDs and chemical agents themselves is less clear, and existing studies in this regard often suffer from methodological challenges and limitations [27]. First, few studies have accounted for robust sets of officer, civilian, and situational factors that may be correlated with both civilian injury and the modality of force used. Second, several studies are based on small samples of police agencies and decades-old data sources, creating concern about the generalizability of these findings to larger populations and current contexts [11, 15–17, 20]. Finally, researchers have often focused on the presence/absence of a documented injury, rather than the severity of the documented injury [6, 15, 20]. Given evidence that many UOF injuries are minor and inconsistently recorded across police agencies [8, 24], it is important to incorporate more objective measures of injury severity.

The present study attempts to address these limitations using administrative data on UOF incidents collected from 17 large urban and metropolitan US law enforcement agencies spread across four geographic regions from 2015 to 2019. Specifically, we test for differential impacts of less-lethal weapons (ECW/CEDs, chemical agents, impact weapons, police canines) on both the likelihood and severity of civilian injury, while controlling for a variety of officer, civilian, and situational characteristics. Conditional upon these characteristics, we also estimate the adjusted probability of civilian injury outcomes for incidents involving each less-lethal weapon type.

## Methods

From August 2021 to May 2023, a total of 99 law enforcement agencies were asked to provide incident-level data on UOF encounters documented between 2015 and 2019. This sampling frame was developed from a set of agencies that previously participated in a nationally representative survey on UOF and officer-involved shootings [28]. To ensure a sufficiently large sample of incidents, eligible agencies were limited to those that reported at least 100 sworn full-time officers and were in at least the 80th percentile for officer-involved shooting incidents. Agencies were asked to provide data on a random sample of up to 200 UOF incidents involving the use of a weapon on a human subject. Agencies with fewer than 200 UOF incidents were asked to provide information on the full population of incidents that occurred during the study period.

In total, 26 agencies from 14 states agreed to participate and provided data on 3707 UOF encounters involving only a less-lethal weapon. The participating agencies consisted of large municipal or county agencies (73% had 250 or more officers) from all regions of the country except the Northeast. De-identified agency characteristics can be found in the Supplementary Material.

The UOF data were provided by agencies in either electronic or hard copy. For hard copy data, coders reviewed each case and manually entered the relevant data into a project database template using a uniform coding scheme. Upon coding, the research staff conducted interrater reliability (IRR) checks to ensure accuracy. Data submitted in an electronic database were cleaned and harmonized by research staff by recoding variables into standardized codes. The UOF encounter data included information on up to two officers and one subject who used (or received) the highest level of force. The highest level of force was coded based on approaches used in prior research [29], prioritizing impact weapons (e.g., less-lethal projectiles, batons), followed by police canines, ECW/CEDs (e.g., TASER), and chemical agents (e.g., pepper spray). If more than two officers used the same level of force, or more than one subject received the same level of force, officers and suspects were randomly selected for coding. These situations were rare, however, as approximately 90% of incidents involved only one subject and two or fewer officers.

Complete information on officer, civilian, and situational characteristics (see below) was not available for all agencies. Our primary specifications focus only on incident-level characteristics that were missing in less than 20% of cases overall. In total, 17 of the 26 agencies met this criterion (i.e., reported some amount of non-missing data on all variables of interest). The 2348 cases provided by these 17 agencies comprise our primary analytic sample. However, we also conducted sensitivity analyses using the full sample of less-lethal force incidents from all 26 agencies (*N* = 3707).

### Statistical Approach

Our dependent variable of interest is the likelihood and severity of civilian injury in each UOF incident. This was coded as a three-item measure consisting of “not injured,” “injured but not hospitalized,” or “injured and hospitalized or killed.” Of note, we combine hospitalizations and fatalities here due to the small number of civilian deaths observed in these encounters (*n* = 3). Our primary independent variable is the weapon type associated with the highest level of force used in each incident (1 = “impact weapon,” 2 = “canine,” 3 = “ECW/CED,” 4 = “chemical agent”), which we treated as nominal to allow for direct contrasts between modalities of less-lethal force. Impact weapons included objects used to strike subjects (e.g., baton, flashlight) as well as less-lethal projectiles (e.g., tear gas, rubber bullets), while chemical agents were generally limited to the use of handheld pepper spray. Incidents that involved multiple less-lethal weapons were coded as only one weapon type; however, we controlled for these incidents in multiple regression models by including the number of weapon types used in each encounter as a covariate (3.5% of incidents).

We also controlled for various officer, civilian, and situational characteristics previously shown to predict UOF decisions and/or civilian injury [7, 15, 16, 30]. These included—among others—the demographic characteristics of the primary officer and civilian involved in the encounter, the rank and assignment of the officer involved, whether the civilian resisted arrest or possessed a weapon, and the underlying reason for the encounter. Finally, we controlled for between-agency differences in the likelihood of injury and trends or shocks across time using agency and year fixed effects. The use of agency fixed effects substantially improved our model fit and variation explained (the latter increased from 32 to 48% across models) but did not affect substantive inferences about weaponry effects. Given the variation in sampling probabilities across agencies, all primary analyses also incorporated sampling weights developed according to the proportion of total cases sampled from each agency [31]. Weighted descriptive statistics for all study variables can be seen in Table 1.

**Table 1:** Weighted incident characteristics (*N* = 2348): 17 police agencies, 2015–2019

	Weighted mean/proportion	Weighted SD
Civilian injury		
Not injured	0.51	-
Injured not hospitalized	0.33	-
Hospitalized or killed	0.16	-
Weapon type		
ECW/CED	0.72	-
Chemical agent	0.09	-
Impact weapon	0.09	-
Canine	0.10	-
Number of weapons used	1.03	0.18
Non-patrol assignment^a^	0.27	-
Sergeant or lieutenant^b^	0.10	-
Female officer	0.08	-
Officer race/ethnicity		
White	0.67	-
Black	0.11	-
Hispanic/other	0.23	-
Two or more officers used force	0.37	-
Civilian resisted	0.80	-
Civilian possessed weapon	0.18	-
Female civilian	0.10	-
Civilian race/ethnicity		
White	0.36	-
Black	0.41	-
Hispanic/other	0.22	-
Civilian age	31.99	10.57
Two or more civilians involved	0.04	-
Reason for encounter		
Unlawful/suspicious activity	0.63	-
Routine patrol	0.12	-
Traffic stop	0.10	-
Warrant	0.03	-
Medical/welfare assistance	0.05	-
Other	0.07	-

^a^Reference = patrol assignment

^b^Reference = officer/deputy/corporal

We modeled the differential effects of less-lethal weapons on civilian injury using multinomial logistic regression, comparing separately the effects of each weapon type on injury without hospitalization (relative to no injury) and injury resulting in hospitalization or death (relative to no injury). In all models, the effects of different weapon types were estimated relative to those of ECW/CED weapons (which were the most commonly used less-lethal weapon). We also checked for potential multicollinearity issues using variance inflation factors and tolerance statistics. Further details of these regression models can be found in the Supplemental Material.

Roughly 30% of incidents in our final analytic sample (*N* = 2348) contained missing data, with the average case missing information from approximately one variable. We used multiple imputation with chained equations to address this missingness, imputing 20 data sets with 5 iterations of each [32]. All models were estimated on each of the 20 imputed data sets, with final coefficients and standard errors pooled using the rules proposed by Rubin [33].

To test the robustness of our findings, we re-estimated our models using only incidents with complete information (i.e., without multiple imputation) from our primary analytic dataset of 2348 cases (*N* = 1653). We also replicated these analyses using all situational covariates included in the full data set, regardless of missingness. This allowed for the inclusion of all 3707 cases from all 26 agencies. We used multiple imputation for missing data in this expanded analysis but note that some agencies required complete imputation of one or more incident-level characteristics. All sensitivity analyses produced results substantively similar to those described below (see Supplementary Material).

All analyses were conducted using the R Project for Statistical Computing [34], and we evaluated the statistical significance of our results using an alpha level of 0.05 and two-tailed significance tests. The project was approved by the Internal Review Board of the National Opinion Research Center.

## Results

Approximately 50% of UOF encounters resulted in a documented civilian injury (Table 1). Minor injuries were twice as likely as those resulting in hospitalization or death. In total, three civilians were killed (0.1%) as a result of less-lethal weapons, with two of these incidents involving the use of an ECW/CED and one involving the use of a police canine. ECW/CEDs were also the most frequently used less-lethal weapon, accounting for over 70% of UOF encounters, while police canines, impact weapons, and chemical agents each accounted for roughly 10% of UOF encounters. Weapon frequencies were similar across racial/ethnic groups, though Black civilians were most likely to have chemical agents used against them (weighted percentage of 14%), White civilians were most likely to have police canines used against them (weighted percentage of 15%), and Hispanic/other civilians were most likely to have impact weapons used against them (weighted percentage of 15%). Racial compositions did not notably differ with respect to ECWs/CED. In bivariate comparisons (Table 2), chemical agents resulted in no civilian injury more often than other weapon types (weighted percentage of 73%) and hospitalization or death less often than other weapon types (weighted percentage of 2%). In contrast, police canines were least likely to result in no civilian injury (weighted percentage of 18%) and most likely to result in hospitalization or death (weighted percentage of 50%).

**Table 2:** Unadjusted probabilities of civilian injury by weapon type

	Weighted proportion		
	No injury	Injury no hosp.	Hosp. or death
ECW/CED	0.56	0.33	0.11
Chemical agent	0.73	0.25	0.02
Impact weapon	0.27	0.50	0.24
Canine	0.18	0.32	0.50
χ^2^	365.50**		

*Hosp.* hospitalization

^**^p < 0.001

Controlling for officer, civilian, and situational characteristics, multinomial regression results indicate that the use of police canines increased the risk of injury without hospitalization by a factor of more than 20 compared to ECW/CEDs (Table 3). Additionally, incidents in which the primary officer worked in a non-patrol assignment (relative to patrol) were associated with a statistically significant 46% reduction in the risk of injury without hospitalization, while civilian weapon possession led to a statistically significant 94% increase in the risk of injury without hospitalization.

**Table 3:** Multinomial regression results for civilian injury and injury severity

	Injured not hospitalized vs. not injured			Injured and hospitalized or killed vs. not injured		
	RRR	95% CI	*p*	RRR	95% CI	*p*
Intercept	**16.91**	**2.85**, **100.32**	** < 0.01**	0.89	0.11, 6.90	0.91
Weapon type^a^						
Chemical agent	1.62	0.76, 3.48	0.21	**0.19**	**0.06**, **0.58**	** < 0.01**
Impact weapon	1.08	0.28, 4.18	0.91	1.45	0.33, 6.40	0.62
Canine	**21.04**	**9.11**, **48.57**	** < 0.01**	**26.5**	**11.19**, **62.75**	** < 0.01**
Number of weapons	1.05	0.35, 3.16	0.94	2.50	0.78, 8.00	0.12
Non-patrol assignment	**0.54**	**0.30**, **0.97**	**0.04**	1.10	0.54, 2.21	0.80
Sergeant or Lieutenant	1.19	0.50, 2.83	0.70	2.06	0.93, 4.56	0.07
Female officer	0.73	0.32, 1.66	0.46	1.25	0.59, 2.66	0.56
Officer race/ethnicity^b^						
Black	1.21	0.60, 2.44	0.59	2.54	0.98, 6.57	0.05
Hispanic/other	1.07	0.52, 2.22	0.85	0.75	0.31, 1.80	0.52
Multiple officers used force	1.07	0.51, 2.24	0.87	1.22	0.55, 2.71	0.62
Civilian resisted	1.51	0.83, 2.77	0.18	0.97	0.44, 2.15	0.94
Civilian possessed weapon	**1.94**	**1.03**, **3.62**	**0.04**	1.84	0.95, 3.58	0.07
Female Civilian	0.47	0.19, 1.20	0.11	1.06	0.43, 2.57	0.90
Civilian race/ethnicity^b^						
Black	1.13	0.63, 2.04	0.68	1.02	0.54, 1.94	0.95
Hispanic/other	1.20	0.64, 2.24	0.56	0.86	0.41, 1.78	0.68
Civilian age	1.01	0.98, 1.03	0.67	1.00	0.97, 1.03	0.90
Two or more civilians involved	0.92	0.12, 7.16	0.94	1.00	0.12, 8.18	1.00
Reason for encounter ^c^						
Routine patrol	1.82	0.83, 3.99	0.14	0.57	0.20, 1.63	0.29
Traffic stop	0.59	0.32, 1.06	0.07	0.92	0.40, 2.09	0.84
Warrant	1.18	0.25, 5.57	0.84	2.23	0.69, 7.24	0.18
Medical/welfare assist	2.24	0.81, 6.21	0.12	1.39	0.54, 3.60	0.50
Other	1.64	0.69, 3.91	0.26	1.03	0.31, 3.45	0.97
Agency fixed effects	Yes					
Year fixed effects	Yes					
*N* of agencies	17					
*N* of observations	2348					
Pooled *D*_2_	26.31 (*p* < 0.01)					
McFadden's *R*^2^	0.48					

*RRR* relative risk ratio. McFadden’s *R*^2^ represents the average *R*^2^ value across each imputed data set. *D*_2_ is a pooled chi-square statistic that is *F*-distributed and compares the full model with an intercept-only model [35]. Estimates are pooled across 20 multiply imputed data sets. Bold values indicate statistical significance at the *p* < 0.05 level

^a^Reference = ECW/CED

^b^Reference = White

^c^Reference = unlawful/suspicious activity

Bold values indicate statistical significance at the *p* < 0.05 level

Police canines were also associated with more severe injuries, significantly increasing the risk of hospitalization or death by a factor of more than 25 relative to ECW/CEDs. Chemical agents, in contrast, led to a statistically significant 81% decrease in the risk of hospitalization or death. No other predictors reached conventional levels of statistical significance in this model.

**Fig. 1:**
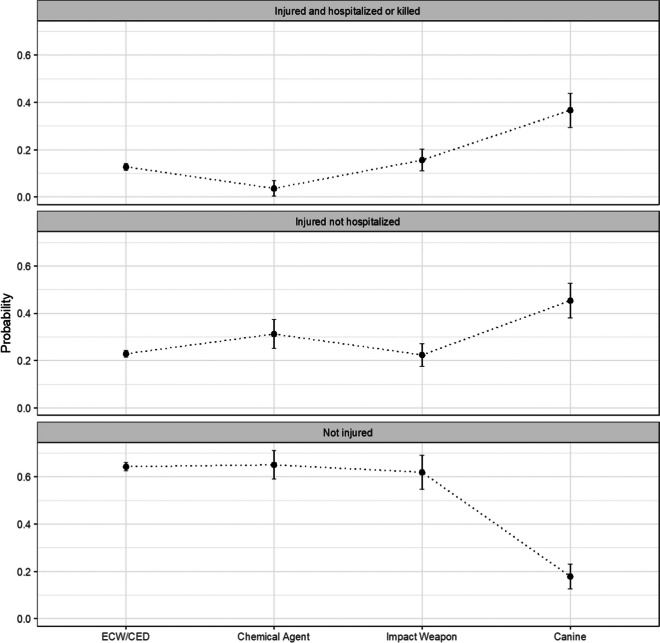
Average counterfactual probabilities for civilian injury outcomes

Based on our regression results, we estimated the average counterfactual probabilities of civilian injury outcomes for each less-lethal weapon type. These predictions are averaged across all complete observations, altering only the less-lethal weapon used in each encounter [36]. As such, these estimates represent predicted injury outcomes if all encounters involved separately the use of each weapon type. As seen in Fig. 1, police canines are predicted to cause civilian injury in 45% of cases (95% CI = 38%, 53%) and hospitalization or death in 37% of cases (95% CI = 29%, 44%), with both estimates being significantly larger than those of all other weapon types. Chemical agents, in contrast, are predicted to cause injury without hospitalization in 31% of cases (95% CI = 25%, 37%) and hospitalization or death in only 4% of cases (95% CI = 0.4%, 7%). The probability of hospitalization or death for chemical agents is significantly smaller than that of both ECW/CEDs (*P* = 13%, 95% CI = 11%, 14%) and impact weapons (*P* = 16%, 95% CI = 11%, 20%).

## Discussion

Using administrative data collected from 17 US law enforcement agencies, spread across the US, covering over 2348 UOF incidents, this study tested the effect of less-lethal weapons on civilian injury and injury severity, conditional on a robust set of officer, civilian, and situational characteristics. Our findings indicate that the use of chemical agents significantly reduced the likelihood of hospitalization or death for civilians involved in UOF encounters, relative to other less-lethal weapons. In contrast, the use of police canines led to significantly larger increases in both the likelihood of civilian injury and the severity of civilian injury, relative to other less-lethal weapons.

Our findings are largely consistent with prior work on less-lethal weapons [6, 11, 15, 17], suggesting that—on average—chemical agents and ECW/CEDs pose the lowest risk to civilians involved in UOF encounters while police canines pose the highest risk. However, the fact that chemical agents reduced the risk of hospitalization or death significantly more than ECW/CEDs in our study should not be understated. Chemical agents and ECW/CEDs are two of the most common less-lethal weapons employed by police agencies. Recent estimates suggest that over 90% of police agencies authorize the use of both weapon types [14]. However, prior research has indicated that there is little consistency as to where these weapon types exist on UOF continuums or force option models, with some agencies considering both to be comparable levels of force [24, 25]. Despite this, chemical agents and ECW/CEDs have distinctly different mechanisms of effect and potential pathways to injury. Whereas chemical agents incapacitate suspects by causing pain and irritation to the eyes and skin [19], ECW/CEDs produce an electric current that causes involuntary muscle contraction [18]. The risk of severe injury for both weapons has generally been linked to interaction with pre-existing health conditions; however, ECW/CEDs also carry the secondary potential for injury resulting from a subsequent fall, or in rare cases, cardiac disturbances related to the electrical shock [37].

Though we cannot pinpoint how civilian injuries occurred, our results suggest that—for civilians directly involved in individual UOF encounters—chemical agents may be less severe forms of force than ECW/CEDs. Our data indicates, however, that the use of ECW/CEDs is currently much more common than the use of chemical agents during UOF encounters involving less lethal weapons. Further, there is substantial variation in the use of ECW/CEDs and chemical agents across agencies and officers, reflecting differences in policies, training, and discretionary practices regarding the use of these weapons. In our sample, the use of ECW/CEDs ranged from a low of 29% to a high of 98% of cases per agency, while the use of chemical agents ranged from a low of 0% to a high of 48% of cases per agency.

An important complementary question is whether there exists a tradeoff between civilian and officer safety when using less-lethal weapons. Though officer injuries in our data were rare (less than 10% of incidents), we note limited support for this hypothesis. In supplementary regression models (see Supplementary Material), we find that police canines significantly reduced the odds of officer injury, but that no significant differences existed between ECW/CEDs, chemical agents, and impact weapons after controlling for officer, civilian, and situational characteristics. In combination with our main findings, these results suggest that increased use of chemical agents—in place of ECW/CEDs or impact weapons—may reduce the risk of civilian injury without jeopardizing officer safety. However, we caution against making global recommendations from these findings for several reasons.

First, we cannot fully account for the context of less-lethal weapon use across the incidents included in our sample. Recent studies have suggested that injury risk and severity from less-lethal weapons—particularly chemical agents and kinetic impact projectiles—may be greater in crowded settings where larger numbers of civilians are exposed [4, 21, 22]. In these contexts, airborne agents and projectiles may strike unintended targets or linger in urban environments [21, 23, 38]. The incidents analyzed in this study primarily represent encounters between police and individual civilians. As such, we cannot speak to the differential risk of injury when less-lethal weapons are deployed as crowd control weapons or in the vicinity of other civilians. This is particularly salient given the widespread use of chemical agents and impact weapons during recent protests across US cities and college campuses [3, 4]. Second, recent studies have pointed to potential long-term or delayed side effects associated with exposure to chemical agents, which are unlikely to be captured in our data [4]. Though it is possible that these secondary effects are more likely following the use of area-wide chemical agents (e.g., tear gas) rather than handheld OC/pepper spray, we cannot definitively test this hypothesis given the current data. Thus, while chemical agents are associated with less severe injuries, it is unclear whether they lead to greater numbers of injuries secondary to the immediate UOF encounter. This potential complicates broad policy conclusions, and more research is needed.

Similar caution should be exercised with regard to police canines, given that their use corresponds to both an increased risk of civilian injury and a decreased risk of officer injury. In our sample, the use of police canines ranged from a low of 0% to a high of 46% of cases per agency, but we lack the agency-level data needed to understand when and why police canines were used. In general, canines are intended for highly specified situations where other types of less-lethal weapons may not be applicable, such as the detection of suspects at night and/or situations in which the perceived threat to officer or public safety is high [39]. As a result of this, some have argued that police canines deescalate encounters and prevent the use of deadly force in situations where it would otherwise be necessary [39, 40]. This is not a hypothesis that we can directly test in our data, and thus, we can only confidently suggest that—in an effort to reduce civilian injury—the use of police canines should be reserved for situations in which they are truly necessary to resolve an encounter.

One of the most important implications of our findings concerns the distinction between injury prevalence and severity. Prior efforts of this kind are often limited to binary measures of injury [6, 15, 20, 23, 41], which may under or overestimate the risks associated with less-lethal weapons. Our results, for example, indicate that hospitalization data alone may severely underestimate the prevalence of civilian injury, and that for certain weapon types, injury may be more likely than not. In contrast, we also find that the majority of injuries associated with certain weapon types (e.g., chemical agents and ECW/CEDs) appear minor, suggesting the importance of considering both outcomes when structuring UOF policies.

Injury prevalence and severity are also important given the potential for police UOF to perpetuate racial and ethnic health inequities. Though we do not find a significant direct effect of race/ethnicity on injury risk, the majority of the civilians included in our sample were non-White, and our results point to aggregate racial/ethnic differences in the frequency with which certain less-lethal weapons are used. Taken together, these results suggest disproportionate consequences of less-lethal weapon usage for certain people and places. UOF policy and practice should take into consideration the immediate risk of less-lethal weapon usage for the individuals involved, but also the broader application of less-lethal weapons across sociodemographic groups and implications for the communities affected [42].

These findings are not without limitations. First, our sample is not nationally representative and intentionally excludes smaller police agencies. Second, we observed significant variation in injury rates across agencies, possibly pointing to inconsistency in the way that agencies defined and recorded these outcomes (despite our efforts at harmonizing the data). Third, our analyses focused on the most severe form of force used in each encounter, and thus, incidents involving multiple forms of force received only one weapon-type code. Though these incidents were rare, and our analyses accounted for the number of weapons used in each encounter, we were unable to identify which weapon was responsible for civilian injury when multiple were used. Our data are also inherently non-experimental and subject to omitted variable bias. It is possible that additional unobserved factors may confound our relationships of interest.

Finally, our study focuses only on the use of less-lethal weapons and their efficacy for reducing civilian injury. As such, we do not consider other forms of force such as hands-on tactics, which are used in the majority of UOF encounters and may account for the majority of civilian injuries [11]. We also do not consider the failure rate of less-lethal weapons or their effectiveness in successfully apprehending civilians. This is an important consideration for law enforcement practitioners, and additional research comparing both injury risk and effectiveness across weapon types is needed. Nonetheless, multi-agency data on civilian injury in UOF encounters is difficult to collect, and this study represents one of the largest and most current analyses of these relationships to date.

## Conclusion

Less-lethal weapons can be effective tools to reduce the risk of civilian injury during UOF encounters in large urban and suburban jurisdictions, but our results suggest that certain modalities of force are significantly more effective in this regard than others. Our findings indicate that police agencies can reduce the severity of civilian injury by adopting policies and training that limit the use of police canines and maximize the use of chemical agents in situations where minor force is sufficient, and that these measures can be taken without jeopardizing officer safety. However, more research is needed on the long-term effects of chemical agents and their risk within group settings. Absent this research, global policy recommendations should be made with caution.

## Supplementary Information

Below is the link to the electronic supplementary material.

Supplementary file1 (DOCX 51 KB)

## Data Availability

The data used in this project is archived on the Open Science Framework (https://osf.io/ju4nw/). R code is available upon request.
