# Supplementary material for: Less-Lethal Weapons and Civilian Injury in Police Use of Force Encounters: A Multi-agency Analysis
Source: J Urban Health. 2024 Nov 11;102(2):389–99. doi: 10.1007/s11524-024-00940-1 (PMC12031711; doi:10.1007/s11524-024-00940-1)
Supplement: Supplementary file 1 — Supplementary file1 (DOCX 51 KB) [file 11524_2024_940_MOESM1_ESM.docx]

**SUPPLEMENTARY MATERIAL FOR**

*“Less-Lethal Weapons and Civilian Injury in Police Use of Force Encounters: A Multi-Agency Analysis”*

**Table of Contents**

[*Agency characteristics 2*](#_Toc164426085)

[*Regression Specifications 3*](#_Toc164426086)

[*Raw regression results for civilian injury: 17 police agencies, 2015-2019 4*](#_Toc164426087)

[*Full imputation regression results for civilian injury: 17 police agencies, 2015-2019 5*](#_Toc164426088)

[*Regression results for officer injury: 17 police agencies, 2015-2019 6*](#_Toc164426089)

[*References 7*](#_Toc164426090)

| **Agency characteristics** | | | | |
| --- | --- | --- | --- | --- |
|  | *N* (%) | Region | *N* of officers | Type |
| **Agency 1** | 157 (4.24) | West | 100-249 | Municipal |
| Agency2 | 179 (4.83) | Midwest | 250 or more | Municipal |
| **Agency3** | 46 (1.24) | West | 100-249 | Municipal |
| Agency 4 | 36 (0.97) | West | 250 or more | County |
| **Agency 5** | 162 (4.37) | Southeast | 250 or more | County |
| Agency 6 | 190 (5.13) | Southeast | 250 or more | County |
| **Agency 7** | 157 (4.24) | Southeast | 250 or more | Municipal |
| Agency 8 | 198 (5.34) | Southeast | 250 or more | County |
| **Agency 9** | 125 (3.37) | Midwest | 100-249 | Municipal |
| **Agency 10** | 197 (5.31) | Southeast | 250 or more | County |
| Agency 11 | 200 (5.4) | West | 250 or more | Municipal |
| Agency 12 | 200 (5.4) | West | 250 or more | Municipal |
| **Agency 13** | 195 (5.26) | West | 250 or more | Municipal |
| Agency 14 | 127 (3.43) | West | 250 or more | County |
| **Agency 15** | 94 (2.54) | South | 250 or more | Municipal |
| Agency 16 | 140 (3.78) | Southeast | 250 or more | Municipal |
| Agency 17 | 169 (4.56) | South | 250 or more | Municipal |
| **Agency 18** | 186 (5.02) | Southeast | 250 or more | County |
| Agency 19 | 196 (5.29) | Southeast | 250 or more | County |
| Agency 20 | 28 (0.76) | Midwest | 100-249 | County |
| Agency 21 | 200 (5.4) | Southeast | 250 or more | County |
| Agency 22 | 150 (4.05) | West | 100-249 | Municipal |
| Agency 23 | 122 (3.29) | Southeast | 100-249 | Municipal |
| Agency 24 | 107 (2.89) | Southeast | 250 or more | County |
| Agency 25 | 104 (2.81) | West | 100-249 | Municipal |
| Agency 26 | 42 (1.13) | Southeast | 250 or more | Municipal |
| *Note:* Bolded agencies were omitted from our main analysis due to complete missingness on covariates | | | | |

## **Regression Specifications**

To assess the effect of less-lethal weapons on civilian injury outcomes we estimated a multinomial logistic regression of the following form:^1^

$${log}_{e}\frac{\pi_{ij}}{\pi_{im}}= \gamma_{0j}+\gamma_{1j}X_{i1} + ...+ \gamma_{kj}X_{ik}$$

Where ${log}_{e}\frac{\pi_{ij}}{\pi_{im}}$ represents the natural log of the probability that the *i*th observation falls within the *j*th category of the dependent variable (e.g., injured but not hospitalized, injured and hospitalized or killed) relative to the baseline category *m* (not injured), $\gamma_{0j}$ represents the intercept for each *j*th category, $\gamma_{1j}$ through $\gamma_{kj}$ represent the regression coefficients for weapon type, officer, civilian, and situational characteristics, and agency and year fixed effects. Model results are reported using relative risk ratios (RRR), which can be converted to a percentage point change in the probability of each injury outcome, relative to no injury, for a one-unit change in $\gamma_{kj}$ as follows [(RRR – 1) *100]. Supplementary analyses examining the effect of less-lethal weapons on officer injury followed a similar approach, however, injuries resulting in hospitalization or death were extremely rare (less than 1%). Thus, we created a dichotomous measure of officer injury (0 = no injury; 1 = injury) and regressed this outcome on officer, civilian, and situational characteristics using a binary logistic regression.

Below, we present the results of our civilian injury models using only complete cases without imputation, followed by the results using larger models that include additional agencies and covariates but with more reliance on imputation. We then present the results of our officer injury model. Of note, we also estimated raw and full imputation models for officer injury. These approaches produced similar results and are available upon request.

| **Raw regression results for civilian injury: 17 police agencies, 2015-2019** | | | | | | |
| --- | --- | --- | --- | --- | --- | --- |
|  | *Injured not hospitalized vs.  Not injured* | | | *Injured and hospitalized or killed vs. Not injured* | | |
|  | RRR | 95% CI | *p* | RRR | 95% CI | *p* |
| Intercept | **15.46** | **1.73, 137.90** | **0.01** | 0.55 | 0.04, 7.97 | 0.66 |
| Weapon type ^a^ |  |  |  |  |  |  |
| Chemical agent | 1.44 | 0.50, 4.08 | 0.50 | **0.06** | **0.02, 0.25** | **< 0.01** |
| Impact weapon | 1.60 | 0.38, 6.70 | 0.52 | 1.43 | 0.26, 7.69 | 0.68 |
| Canine | **28.68** | **10.7, 76.84** | **< 0.01** | **30.80** | **10.76, 88.12** | **< 0.01** |
| Number of weapons | 1.05 | 0.32, 3.47 | 0.93 | 2.97 | 0.75, 11.79 | 0.12 |
| Non-patrol assignment | **0.41** | **0.22, 0.79** | **0.01** | 0.90 | 0.40, 2.03 | 0.81 |
| Sergeant or Lieutenant | 0.93 | 0.28, 3.11 | 0.90 | 1.84 | 0.71, 4.73 | 0.21 |
| Female officer | 0.53 | 0.17, 1.61 | 0.26 | 0.92 | 0.39, 2.17 | 0.85 |
| Officer race/ethnicity ^b^ |  |  |  |  |  |  |
| Black | 0.90 | 0.45, 1.80 | 0.77 | **3.39** | **1.07, 10.75** | **0.04** |
| Hispanic/Other | 1.17 | 0.52, 2.61 | 0.70 | 0.85 | 0.31, 2.31 | 0.74 |
| Multiple officers used force | 1.09 | 0.49, 2.41 | 0.84 | 1.38 | 0.57, 3.35 | 0.48 |
| Civilian resisted | 1.74 | 0.93, 3.25 | 0.08 | 0.97 | 0.39, 2.41 | 0.95 |
| Civilian possessed weapon | 1.90 | 0.92, 3.91 | 0.08 | 1.56 | 0.70, 3.48 | 0.28 |
| Female Civilian | 0.50 | 0.20, 1.27 | 0.15 | 1.20 | 0.44, 3.27 | 0.72 |
| Civilian race/ethnicity ^b^ |  |  |  |  |  |  |
| Black | 1.35 | 0.69, 2.64 | 0.39 | 1.28 | 0.64, 2.55 | 0.48 |
| Hispanic/Other | 0.97 | 0.44, 2.16 | 0.94 | 1.03 | 0.43, 2.45 | 0.95 |
| Civilian age | 1.01 | 0.99, 1.04 | 0.35 | 1.01 | 0.98, 1.04 | 0.48 |
| Two or more civilians involved | 0.39 | 0.02, 6.98 | 0.52 | 0.47 | 0.01, 17.08 | 0.68 |
| Reason for encounter ^c^ |  |  |  |  |  |  |
| Routine patrol | 1.65 | 0.70, 3.89 | 0.25 | 0.56 | 0.18, 1.74 | 0.32 |
| Traffic stop | **0.51** | **0.27, 0.97** | **0.04** | 0.74 | 0.29, 1.88 | 0.53 |
| Warrant | 0.92 | 0.12, 7.06 | 0.94 | 2.07 | 0.61, 7.11 | 0.25 |
| Medical/welfare assist | 2.26 | 0.78, 6.57 | 0.13 | 1.64 | 0.54, 4.96 | 0.38 |
| Other | 1.53 | 0.53, 4.43 | 0.43 | 0.83 | 0.18, 3.88 | 0.81 |
| Agency fixed effects | YES | | | | | |
| Year fixed effects | YES | | | | | |
| *N* of agencies | 17 | | | | | |
| *N* of observations | 1,653 | | | | | |
| Model χ^2^ | 1,425 (*p* < 0.01) | | | | | |
| McFadden's *R*^2^ | 0.44 | | | | | |
| *Note:* RRR = Relative risk ratio. Bold values indicate statistical significance at the *p* < 0.05 level. ^a^ Reference = ECW/CED ^b^ Reference = White ^c^ Reference = Unlawful/suspicious activity | | | | | | |

| **Full imputation regression results for civilian injury: 26 police agencies, 2015-2019** | | | | | | |
| --- | --- | --- | --- | --- | --- | --- |
|  | *Injured not hospitalized vs.  Not injured* | | | *Injured and hospitalized or killed vs. Not injured* | | |
|  | RRR | 95% CI | *p* | RRR | 95% CI | *p* |
| Intercept | **18.66** | **2.21, 157.51** | **0.01** | 0.40 | 0.04, 3.93 | 0.44 |
| Weapon type ^a^ |  |  |  |  |  |  |
| Chemical agent | 1.64 | 0.89, 3.02 | 0.12 | **0.31** | **0.15, 0.65** | **< 0.01** |
| Impact weapon | 1.11 | 0.44, 2.81 | 0.83 | 1.38 | 0.54, 3.51 | 0.50 |
| Canine | **22.53** | **10.77, 47.16** | **< 0.01** | **44.77** | **20.05, 99.96** | **< 0.01** |
| Number of weapons | 1.29 | 0.50, 3.31 | 0.60 | **2.86** | **1.11, 7.39** | **0.03** |
| Non-patrol assignment | 0.65 | 0.37, 1.13 | 0.12 | 1.29 | 0.66, 2.51 | 0.45 |
| Sergeant or Lieutenant | 1.39 | 0.71, 2.74 | 0.34 | **2.00** | **1.01, 3.96** | **0.05** |
| Officer age | 0.99 | 0.96, 1.01 | 0.28 | 0.98 | 0.95, 1.01 | 0.18 |
| Female officer | 0.69 | 0.36, 1.31 | 0.26 | 0.97 | 0.57, 1.67 | 0.92 |
| Officer race/ethnicity ^b^ |  |  |  |  |  |  |
| Black | 1.15 | 0.63, 2.08 | 0.66 | 1.65 | 0.83, 3.26 | 0.15 |
| Hispanic/Other | 1.26 | 0.74, 2.13 | 0.39 | 0.93 | 0.55, 1.57 | 0.78 |
| Multiple officers used force | 1.21 | 0.65, 2.25 | 0.55 | 1.26 | 0.66, 2.39 | 0.48 |
| Civilian used force | 1.14 | 0.74, 1.76 | 0.54 | 1.18 | 0.74, 1.89 | 0.49 |
| Civilian resisted | 1.62 | 0.91, 2.87 | 0.10 | 1.05 | 0.51, 2.20 | 0.89 |
| Civilian possessed weapon | **2.02** | **1.19, 3.45** | **0.01** | **1.73** | **1.00, 2.99** | **0.05** |
| Civilian fled | 1.61 | 0.96, 2.69 | 0.07 | 1.70 | 0.92, 3.13 | 0.09 |
| Civilian mental health crisis | 0.63 | 0.31, 1.30 | 0.21 | 1.69 | 0.8, 3.59 | 0.17 |
| Civilian drug/alc. use | 0.83 | 0.5, 1.38 | 0.48 | 1.38 | 0.86, 2.2 | 0.18 |
| Female Civilian | 0.64 | 0.29, 1.39 | 0.26 | 1.25 | 0.63, 2.5 | 0.52 |
| Civilian race/ethnicity ^b^ |  |  |  |  |  |  |
| Black | 0.89 | 0.52, 1.51 | 0.66 | 1.08 | 0.64, 1.83 | 0.78 |
| Hispanic/Other | 1.14 | 0.66, 1.97 | 0.64 | 0.98 | 0.56, 1.72 | 0.94 |
| Civilian age | 1.01 | 0.99, 1.03 | 0.61 | 1.00 | 0.98, 1.02 | 0.96 |
| Two or more civilians involved | 0.78 | 0.16, 3.87 | 0.76 | 0.63 | 0.15, 2.65 | 0.53 |
| Offense type ^c^ |  |  |  |  |  |  |
| Part I property | 0.96 | 0.48, 1.91 | 0.90 | 0.94 | 0.46, 1.92 | 0.87 |
| Part I violent | 0.94 | 0.59, 1.50 | 0.81 | 1.38 | 0.85, 2.23 | 0.19 |
| Reason for encounter ^d^ |  |  |  |  |  |  |
| Routine patrol | 1.72 | 0.88, 3.39 | 0.12 | 0.88 | 0.4, 1.90 | 0.74 |
| Traffic stop | **0.55** | **0.31, 0.97** | **0.04** | 1.03 | 0.48, 2.21 | 0.94 |
| Warrant | 1.25 | 0.43, 3.62 | 0.68 | 1.85 | 0.63, 5.41 | 0.26 |
| Medical/welfare assist | 1.97 | 0.76, 5.11 | 0.16 | 1.13 | 0.47, 2.71 | 0.79 |
| Other | 1.38 | 0.56, 3.37 | 0.48 | 1.02 | 0.35, 2.99 | 0.97 |
| BWC present | 0.57 | 0.31, 1.04 | 0.07 | 0.84 | 0.41, 1.69 | 0.62 |
| Backup requested | 0.54 | 0.30, 0.98 | 0.04 | 0.63 | 0.31, 1.25 | 0.18 |
| Agency fixed effects | YES | | | | | |
| Year fixed effects | YES | | | | | |
| *N* of agencies | 26 | | | | | |
| *N* of observations | 3,707 | | | | | |
| Pooled *D*_2_ | 29.15 (*p* < 0.01) | | | | | |
| McFadden's *R*^2^ | 0.49 | | | | | |
| *Note:* RRR = RRR = Relative risk ratio. McFadden’s *R*^2^ represents the average *R*^2^ value across each imputed data set. *D*_2_ is a pooled chi-square statistic that is *F*-distributed and compares the full model with an intercept-only model.^2^ Estimates are pooled across 20 multiply imputed data sets. Bold values indicate statistical significance at the *p* < 0.05 level. ^a^ Reference = ECW/CED ^b^ Reference = White  ^c^ Reference = Part II crime ^d^ Reference = Unlawful/suspicious activity | | | | | | |

| **Regression results for officer injury: 17 police agencies, 2015-2019** | | | |
| --- | --- | --- | --- |
|  | OR | 95% CI | *p* |
| Intercept | 0.14 | 0.02, 0.96 | 0.04 |
| Weapon type ^a^ |  |  |  |
| Chemical agent | 0.79 | 0.33, 1.86 | 0.59 |
| Impact weapon | 0.38 | 0.13, 1.08 | 0.07 |
| Canine | **0.04** | **0.01, 0.17** | **< 0.01** |
| Number of weapons | 1.59 | 0.46, 5.45 | 0.46 |
| Non-patrol assignment | 1.17 | 0.53, 2.56 | 0.70 |
| Sergeant or Lieutenant | 0.37 | 0.12, 1.09 | 0.07 |
| Female officer | 0.98 | 0.43, 2.21 | 0.96 |
| Officer race/ethnicity ^b^ |  |  |  |
| Black | 1.03 | 0.46, 2.32 | 0.94 |
| Hispanic/Other | 1.31 | 0.72, 2.37 | 0.38 |
| Multiple officers used force | 1.73 | 0.88, 3.43 | 0.11 |
| Citizen resisted | **1.82** | **1.02, 3.24** | **0.04** |
| Citizen possessed weapon | 0.79 | 0.37, 1.69 | 0.55 |
| Female citizen | 0.93 | 0.41, 2.12 | 0.86 |
| Citizen race/ethnicity ^b^ |  |  |  |
| Black | 0.90 | 0.52, 1.57 | 0.71 |
| Hispanic/Other | **0.45** | **0.22, 0.93** | **0.03** |
| Citizen age | 0.98 | 0.95, 1.00 | 0.06 |
| Two or more civilians involved | 0.81 | 0.23, 2.86 | 0.74 |
| Reason for encounter ^c^ |  |  |  |
| Routine patrol | 0.99 | 0.45, 2.17 | 0.98 |
| Traffic stop | 1.27 | 0.43, 3.73 | 0.66 |
| Warrant | 0.95 | 0.40, 2.21 | 0.90 |
| Medical/welfare assist | **0.22** | **0.09, 0.56** | **< 0.01** |
| Other | 0.71 | 0.25, 2.06 | 0.53 |
| Agency fixed effects | YES | | |
| Year fixed effects | YES | | |
| *N* of agencies | 17 | | |
| *N* of observations | 2,348 | | |
| Pooled *D*_2_ | 3.65 (*p* < 0.01) | | |
| McFadden's *R*^2^ | 0.11 | | |
| *Note:* OR = Odds ratio. McFadden’s *R*^2^ represents the average *R*^2^ value across each imputed data set. *D*_2_ is a pooled chi-square statistic that is *F*-distributed and compares the full model with an intercept-only model.^2^ Estimates are pooled across 20 multiply imputed data sets. Bold values indicate statistical significance at the *p* < 0.05 level. ^a^ Reference = ECW/CED ^b^ Reference = White ^c^ Reference = Unlawful/suspicious activity | | | |

## **References**

1. Fox J. *Applied Regression Analysis and Generalized Linear Models*. Sage; 2016.
2. Robitzsch A, Grund S, Henke T. miceadds: Some Additional Multiple Imputation Functions, Especially for “mice.” R-Packages. 2023. Accessed April 19, 2024. https://cran.r-project.org/package=miceadds
